# Oridonin Prolongs the Survival of Mouse Cardiac Allografts by Attenuating the NF-κB/NLRP3 Pathway

**DOI:** 10.3389/fimmu.2021.719574

**Published:** 2021-09-10

**Authors:** Xiaoxiao Du, Weitao Que, Xin Hu, Xiao Yu, Wen-Zhi Guo, Shuijun Zhang, Xiao-Kang Li

**Affiliations:** ^1^Henan Key Laboratory of Digestive Organ Transplantation, Open and Key Laboratory of Hepatobiliary and Pancreatic Surgery and Digestive Organ Transplantation at Henan Universities, Zhengzhou Key Laboratory of Hepatobiliary and Pancreatic Diseases and Organ Transplantation, Department of Hepatobiliary and Pancreatic Surgery, The First Affiliated Hospital of Zhengzhou University, Zhengzhou, China; ^2^Division of Transplantation Immunology, National Research Institute for Child Health and Development, Tokyo, Japan

**Keywords:** oridonin, cardiac transplantation, NLRP3 inflammasome, acute rejection (AR), adaptive immune, BMDC, NF-κB, Th1 differentiation

## Abstract

**Background:**

Oridonin (Ori), the main bioactive ingredient of the natural anti-inflammatory herb *Rabdosia rubescens*, could be a covalent inhibitor of the NLRP3 inflammasome. Solid organ transplantation provides a life-saving optional therapy for patients with end-stage organ dysfunction. The long-term survival of solid organ transplantation remains restricted because of the possibility of rejection and the toxicity, infection, cardiovascular disease, and malignancy related to immunosuppressive (IS) drugs. However, the pathogenic mechanisms involved remain unclear. The ideal IS drugs to prevent allograft rejection have not been identified. Here, we investigated whether Ori could prolong the *in vivo* survival of completely mismatched cardiac allografts.

**Methods:**

The cardiac transplantation models were conducted among three groups of mice from C57BL/6NCrSlc (B6/N) or C3H/HeNSlc (C3H) to C3H: the syngeneic and the allogeneic group, whose recipients were treated with vehicle of Ori, and the Ori treatment group, in which the recipients were transplanted hearts from MHC-I mismatched donors and treated with different dosages of Ori from post-operative day (POD) 0 to 7. Then, we investigated the effect of Ori on bone marrow-derived dendritic cell (BMDC) and allogeneic mixed lymphocyte reaction *in vitro*.

**Results:**

Ori with 3, 10, and 15 mg/kg Ori could prolong the survival (MST = 22.8, 49.2, and 65.3 days, respectively). We found that infiltrating CD8^+^ T cells and macrophages were decreased, and regulatory T cells (Tregs) were expanded in allografts on POD7. The mRNA level of IL-1β and IFN-γ of allografts was downregulated. Mechanistically, Ori-treated BMDCs suppressed T-cell proliferation and IFN-γ^+^CD4^+^ T-cell differentiation, along with the expansion of Tregs and IL-10^+^CD4^+^ T cells. Ori inhibited NOD, LRR-, and pyrin domain-containing protein 3 (NLRP3) expression; attenuated NF-κB and IκBα phosphorylation in LPS-activated BMDCs; downregulated NLRP3, Caspase-1, IL-1β, IL-18, and IFN-γ; and upregulated IL-10 expression.

**Conclusions:**

Our findings highlight the potential of Ori as a novel and natural IS agent to improve transplant tolerance. Ori could exert IS activity through decreasing IL-1β and IL-18 production and Th1 differentiation and proliferation and expanding Tregs *via* inhibiting the NF-κB/NLRP3 signaling pathway.

## Introduction

Oridonin (Ori), a natural ent-kaurane diterpenoid, is the major bioactive ingredient of *Rabdosia rubescens*, which has been widely applied in traditional Chinese and Japanese medicine for multiple diseases (1, 2). Since Ori was extracted out and its structure was confirmed in 1970 (3), many researchers have explored a variety of its functions and mechanisms, focusing on its anticancer activities such as the induction of cell cycle arrest, apoptosis, and angiogenesis suppression in various cancers (4–8). Alternatively, Ori also exerts considerable anti-inflammatory activity by inhibiting the activation of NF-κB and/or MAPK, resulting in the suppression of the release of proinflammatory cytokines including interleukin (IL)-6 and tumor necrosis factor (TNF)-α (9–11) in colitis, sepsis, and neuroinflammation (12–15).

Recently, Ori was reported as a specific covalent inhibitor of NOD, LRR-, and pyrin domain-containing protein 3 (NLRP3) inflammatory bodies, which can bind to cysteine 79 of the nucleotide-binding oligomerization domain (NACHT) through covalent bond formation, to prevent NIMA (never in mitosis gene a)-associated expressed kinase 7 (NEK7)–NLRP3 interaction and subsequent NLRP3 inflammasome activation (16). Since the inflammasome complex was confirmed in 2002 (17), the NLRP3 inflammasome has been extensively studied and consists of NLRP3, adapter protein apoptosis-related speck-like protein (ASC), and caspase-1 (18). NLRP3 is a cytoplasmic sensor in immune cells, like dendritic cells (DCs), macrophages, and T cells (19), and can detect a variety of stimuli signals, including danger-associated molecular patterns (DAMPs) and pathogen-associated molecular patterns (PAMPs), important participants in innate and adaptive immune responses (20, 21). The subsequent NLRP3 inflammasome assembly and activation (19, 22) promotes autophagy, gasdermin D-associated pyroptosis (23, 24), and cleavage of pro-IL-1β and pro-IL-18 to form functional IL-1β and IL-18 (25). This induces pyroptotic cell death (23) and reduces T-cell proliferation (26) and helper T (Th) cell differentiation (21, 27, 28).

Dysregulated NLRP3 inflammasome activity contributes to several inflammatory (29) and autoimmune (30) diseases, graft-*versus*-host disease (GvHD) (31, 32), and corneal, skin allograft rejection (26, 33). Solid-organ allotransplantation rejection also comprises the massive innate and adaptive immune response regulated by IL-1β and IL-18 (34). However, the potential effect and mechanism of the NLRP3 inflammasome remain unclear. Solid-organ transplantation provides a life-saving optional therapy for patients with end-stage organ dysfunction. With the development of immunosuppressive (IS) protocols and surgery skills, the survival of allografts has improved greatly over the past several decades (35). However, the occurrence of acute or chronic allograft rejection and the side effects of IS, such as toxicity, infection, cardiovascular disease, and malignancy, continue to limit the development of organ transplantation greatly (36).

Ori is preventative and therapeutic in mouse models of peritonitis, type 2 diabetes (T2D), and gouty arthritis, *via* blocking NLRP3 activation (16). Our previous research showed that Ori inhibits the proliferation of T cells in a mouse skin transplant model (37). Here, we investigated the potential effects of Ori on the pathogenesis of acute allograft rejection in a mouse cardiac transplant model and the role of NLRP3 inflammasome in the underlying mechanism.

## Materials and Methods

### Animals and Cardiac Transplantation Model

C57BL/6NCrSlc (B6/N, H-2k^b^) and C3H/HeNSlc (C3H, H-2k^k^) mice (male, 7–12 weeks old) were purchased from SLC (Shizuoka, Japan) and raised under specific-pathogen-free conditions according to the guidelines of the Animal Use and Care Committee of the National Research Institute for Child Health and Development, Tokyo, Japan. All animal experimental protocols were approved by the Committee on the Ethics of Animal Experiments of the Institute (Permission Number: A2009-010). The cardiac transplantation operations were conducted as previously described (38, 39). Fully vascularized hearts from donor C3H (syngeneic group) or B6/N (allogeneic and treatment groups) mice were heterotopically transplanted to the abdomen of recipient C3H. The graft survival was identified by palpating the recipient’s abdomen daily. Unpalpable beating was considered cardiac graft rejection and determined visually by laparotomy.

### Reagents and Treatment Protocol

Ori was purchased from Chengdu Yuanye Bio-Technology Co., Ltd. (Chengdu, China). Its purity was greater than 98% (HPLC). Heterotopic heart transplant recipients were divided into three groups: the syngeneic, allogeneic, and Ori-treatment allogeneic groups. The syngeneic and allogeneic groups were treated with vehicle of Ori. In the Ori treatment group, mice were administrated with intraperitoneal injection of Ori (3, 10, and 15 mg/kg/day) from post-operative day (POD) 0 to 7 consecutively. Mouse was sacrificed if the graft was rejected, or the survival of the graft reached the predetermined endpoint on POD100.

### The Culture of Conventional Bone Marrow-Derived DCs and the Administration of Ori

Bone marrow cells (BMCs) were flushed from the femur and tibia of B6/N mice. For the differentiation of conventional BMDCs, after lysing the red cells, BMCs were cultured in complete medium with GM-CSF (10 ng/ml) and IL-4 (10 ng/ml) (PeproTech, Cranbury, NJ) in 24-well tissue culture plates (Greiner Bio-One Japan, Tokyo, Japan). On the second day, the suspension cells were drained away, and half of the medium was refreshed with the same concentration of GM-CSF and IL-4. On day 5, the non-adherent cells were harvested and then reseeded in fresh medium with the same cytokines. For maturation, BMDCs were stimulated with 10 ng/ml LPS (Sigma-Aldrich) for 2 days, considered as immature DC (imDC). With the presence of LPS, different concentrations of Ori (1, 3, and 10 μM) were added in the medium. The group without any treatment was regarded as imDC. On day 7, different groups of non-adherent and suspending cells were harvested for the subsequent experiments and analysis.

### Allogeneic Mixed Lymphocyte Reaction

In the one-way mixed lymphocyte reaction (MLR), nylon-wool column (Wako)-enriched T cells from C3H mice spleen were used as responders and labeled with CellTrace CFSE (Thermo Fisher). Groups of BMDCs above were irradiated with 20 Gy x-ray and used as stimulators. The stimulator BMDCs (2 × 10^4^) and responder T cells (2 × 10^5^) were cocultured in a 96-well U bottom plate within 200 μl/well of complete RPMI medium at 37°C for 5 days. At the end of the culture, cells were collected, stained with fluorescent-labeled antibodies, and analyzed by flow cytometry.

### Supplementary Materials and Methods

Additional materials and methods including the isolation of graft infiltrating lymphocytes, intracellular cytokine staining, flow cytometry analysis, histological analysis, immunohistochemistry, quantitative real time RT-PCR, and Western blotting are accessible in the Supplementary Materials and Methods.

### Statistical Analysis

Statistical analyses were performed with GraphPad Prism 8 (GraphPad Prism Software Inc., San Diego, CA). The survival of cardiac grafts was analyzed with Kaplan–Meier curves and the log-rank tests were applied to compare the differences. Student’s *t*-test was used to compare the means between two groups and one-way ANOVA was used to compare the means among multiple groups. *p*-values of <0.05 were considered to indicate statistical significance.

## Results

### Ori Treatment Prolongs Cardiac Allograft Survival and Decreases Inflammatory Cell Infiltration

**Figure 1:**
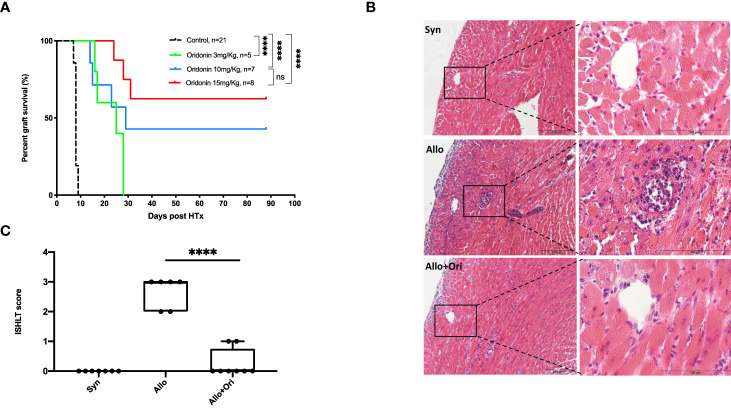
The effect of the Ori on graft survival and pathology in a fully mismatched heart transplantation model. **(A)** The statistical analysis of graft survival of groups with different dosages of Oridonin and allogeneic control with vehicle. ****p < 0.0001, ns, not significant. **(B)** H&E staining of cardiac grafts in syngeneic (Syn), allogenic (Allo), and Ori (Allo + Ori) recipients on POD7. Scale bars represent 100 and 200 µm. **(C)** According to the standard of ISHLT, evaluate the score of graft rejection among groups. Values are shown as the mean ± SD; ****p < 0.0001; NS, not significant.

To assess the potential application of Ori upon the prevention of acute allograft rejection, we conducted heterotopic cardiac transplantation from donor B6/N mice to recipient C3H mice. As shown in **Figure 1A**, compared to the control group with a mean survival time (MST) of 8 days, treatment with different dosages of Ori prolonged the cardiac allograft acceptance in the Ori 3 mg/kg group with an MST of 22.8 days (*p* < 0.0001), Ori 10 mg/kg group with an MST of 49.2 days (*p* < 0.0001), and Ori 15 mg/kg group with an MST of 65.3 days (*p* < 0.0001). We addressed our main aim to explore the potential effect of Ori 15 mg/kg in the subsequent experiments. Consistently, histology showed obvious inflammatory cell infiltration around the coronary arteries in control allografts, whereas they were markedly decreased in the Ori-treated and syngeneic grafts (**Figure 1B**) on POD7. Subsequently, according to the ISHLT grading-score system, the grafts from the control group showed high levels of inflammatory infiltration and moderate or severe myocyte damage (scores ranged from 2 to 3), whereas all isografts scored 0 and Ori-treated grafts scored 0–1 (*p* < 0.0001). These results demonstrated that Ori protected against acute *in vivo* allograft rejection.

### Ori Treatment Reduces T-Cell and Macrophage Recruitment Into Cardiac Allografts

**Figure 2:**
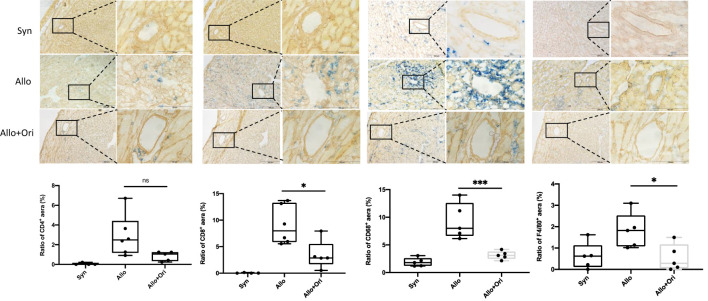
The effect of Ori on the infiltration of T cells and macrophage in cardiac allografts. Cardiac grafts in syngeneic (Syn), allogenic (Allo), and Ori (Allo + Ori) recipients on POD7 were harvested and stained with anti-CD4, CD8, CD68, and F4/80 (blue) separately and collagen IV (yellowish-brown) by immunohistochemistry staining. Magnification, ×100 and ×400; scale bars represent 100 and 200 µm. The positive aera ratio was analyzed and calculated from five arbitrarily selected fields of view. Values are shown as the mean ± SD; *p < 0.05, ***p < 0.001; NS, not significant.

We used immunohistochemistry for CD4, CD8, CD68, and F4/80 staining to characterize the types of infiltrating immune cells present on POD7. As displayed in **Figure 2**, the myocardial infiltration of CD8^+^ T cells and CD68^+^ or F4/80^+^ macrophages in allografts were significantly decreased in the Ori-treated group compared with the control group on POD7. Semiquantitative analysis confirmed the significant reduction in degree of CD8^+^ T-cell (*p* < 0.05), CD68^+^ (*p* < 0.001), and F4/80 macrophage (*p* < 0.05) infiltration. However, there were no significant differences in the infiltration of CD4^+^ T cells among the Ori-treated and control groups (*p* > 0.05). This suggested that Ori treatment reduced cytotoxic T-cell and macrophage recruitment into cardiac allografts.

### Ori Treatment Reduces the Proportion of CD8^+^ T Cells in Graft-Infiltrating Lymphocytes and Spleens and Increases Tregs in Spleens

**Figure 3:**
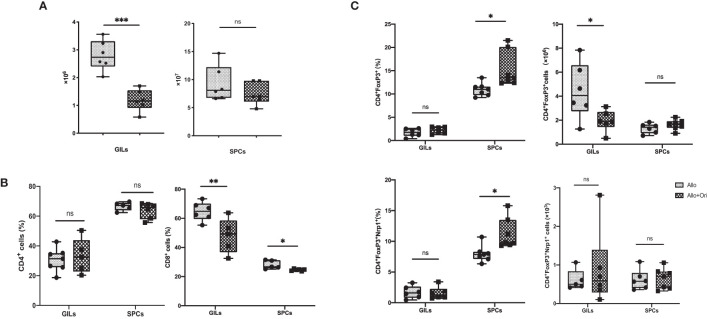
The management of Ori induced alternation of the infiltrating T cells and Treg in GILs and SPCs. Spleens and cardiac grafts were harvested on POD7 from vehicle control (Allo) and Ori-treated (Allo + Ori) groups. SPCs and GILs were isolated, multiply stained with mAb, and then assessed by FCM. Stained GILs and SPCs were gated on lymphocyte live cells. Gating strategy is shown in **Supplementary Figures S1, S2**. **(A)** The total cell numbers of the SPCs and GILs in Allo and Allo + Ori groups. **(B)** The frequencies and absolute numbers of CD4^+^ and CD8^+^ T cells in SPCs and GILs from Allo and Allo + Ori groups. **(C)** The frequencies and absolute numbers of Tregs (CD4^+^FoxP3^+^and CD4^+^FoxP3^+^Nrp1^+^ T cells) in SPCs and GILs from Allo and Allo + Ori groups. Values are shown as the mean ± SD; *p < 0.05, **p < 0.01, ***p < 0.001, ns, no significant.

To clarify the variation of the number and/or phenotype of immune cells after Ori treatment, we performed the FCM analysis with graft-infiltrating lymphocytes (GILs) and spleen cells (SPCs) on POD7. The absolute lymphocyte numbers isolated from Ori-treated allografts were significantly downregulated compared to those in the control group (*p* < 0.001), whereas the change in the spleen was not significant (*p* > 0.05; **Figure 3A**). Both GILs and SPCs in the Ori-treated group had an apparently decreased percentage of CD8^+^ T cells compared with the control group, with the same tendency of the absolute count of CD8^+^ T cells in GILs, but not in SPCs (*p* < 0.01; *p* < 0.05; **Figure 3B**, lower). Although there was no significant variation about the percentage of CD4^+^ T cells in GILs and SPCs, the total CD4^+^ T-cell counts in GILs declined obviously in the Ori-treated group (*p* < 0.001; **Figure 3B**, upper). The percentage of CD4^+^FoxP3^+^ Tregs markedly increased in the spleens from the Ori-treated group (*p* < 0.05; **Figure 3C**, upper). No changes were seen in the total Treg counts or the percentage of CD4^+^FoxP3^+^ Tregs in GILs (*p* > 0.05). The total Treg cell numbers were lower than those of the control group (*p* < 0.05), likely due to the marked difference in the total number of cells. Neuropilin-1 (Nrp-1) is expressed at high levels on natural Tregs from the thymus and is responsible for the immunoregulatory activity of Tregs (40). We found that the percentage of Nrp1^+^ Tregs was significantly increased in the spleen from the Ori-treated group (**Figure 3C**, left, lower). Neither the absolute cell counts nor the Nrp1^+^ Tregs in GILs were significantly different. We also noticed that the proportions of macrophages were downregulated remarkedly in GILs and SPCs (**Supplementary Figures S1, S2**). Additionally, the T-cell subset as shown in **Supplementary Figure S3**, central memory CD4^+^ T cells (CD62L^+^CD44^+^), and resident memory CD8^+^ T cells (CD69^+^CD103^+^) were decreased significantly in spleens. However, there were no significant changes in T-cell subsets in GILs (**Supplementary Figure S4**). We demonstrated that Ori treatment reduced the total inflammatory infiltration and the proportion of CD8^+^ T cells in GILs and increased the percentage of Tregs in spleens.

### IL-1β and Caspase-1 Were Significantly Downregulated in Ori-Treated Allografts

**Figure 4:**
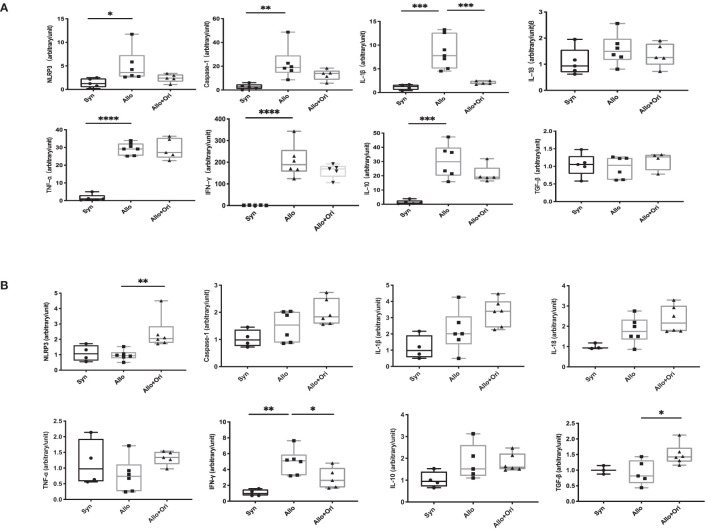
The quantitative changes of mRNA expression in cardiac graft and SPCs following Ori treatment. Cardiac grafts and spleens were harvested on POD7 from syngeneic (Syn), allogenic (Allo), and Ori-treated (Allo + Ori) groups. The total mRNA was extracted from cardiac grafts **(A)** and SPCs **(B)**, and the mRNA expressions of NLRP3, Caspase-1, IL-1β, IL-18, TNF-α, IFN-γ, IL-10, and TGF-β were measured by qRT-PCR. Values are shown as the mean ± SD; *p < 0.05, **p < 0.01, ***p < 0.001, ****p < 0.0001.

To provide further insight into the mechanism underlying the prolonged allograft survival induced by Ori treatment, we performed an analysis of the gene expression of NLRP3 and related cytokines in allografts and spleens on POD7 using quantitative RT-PCR. Notably, the levels of both IL-1β and Caspase-1 were significantly downregulated in Ori-treated allografts from the control group (**Figure 4A**). There was no significant difference among the NLRP3, IL-18, TNF-α, IFN-γ, IL-10, and TGF-β levels in allografts. Moreover, the levels of the proinflammatory cytokine IFN-γ were lower and the levels of the anti-inflammatory cytokine TGF-β were higher in the splenocytes than in the control group (**Figure 4B**). This indicated that Ori treatment may lead to the decrease of IL-1β and Caspase-1, downstream of NLRP3 in grafts, accompanied by the upregulation of TGF-β and downregulation of IFN-γ in the spleen, contributing to the long survival of allografts.

### Ori Treatment Inhibits BMDC Maturation *In Vitro*

**Figure 5:**
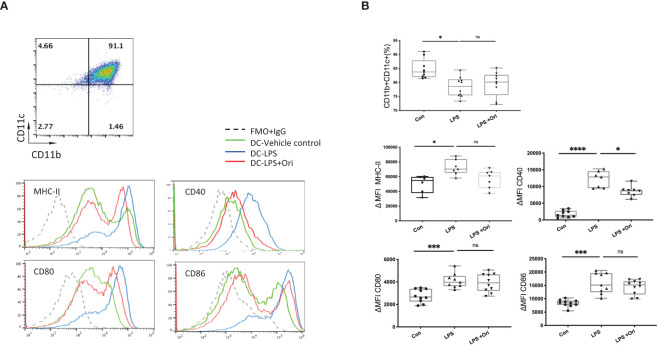
Ori treatment affected the percentage and phenotypes of BMDCs *in vitro*. **(A)** Representative FCM analysis of BMDCs (CD11b^+^CD11c^+^) and the histogram graphs of the MHC-II, CD40, CD80, and CD86 on the gated CD11b^+^CD11c^+^ population of different groups are shown. **(B)** The percentages of the CD11b^+^CD11c^+^ population and the median fluorescence intensity (ΔMFI) of MHC-II, CD40, CD80, and CD86 of BMDCs in each group were calculated. ΔMFI was subtracted from the MFI value of the Isotype control of each antibody of the fluorescence intensity. Values are shown as the mean ± SD; *p < 0.05, ***p < 0.001, ****p < 0.0001, ns, not significant.

DCs are the most important antigen-presenting cells for the differentiation of naïve T cells into their specific subsets (41). To further evaluate whether Ori influences the maturation state of DCs, we assessed the effects of Ori on BMDC maturation *in vitro.* As shown in **Figure 5A**, BMDCs were determined as CD11b^+^CD11c^+^ population cells. Subsequently, the median fluorescence intensities (MFI) of costimulatory molecules, such as MHC-II, CD40, CD80, and CD86, were compared to assess the maturation state of DCs. We found that the purity of DCs activated with LPS was reduced and the ΔMFIs of costimulatory molecules such as MHC-II, CD40, CD80, and CD86 were elevated markedly (**Figure 5B**) compared with imDC, indicating that LPS activated DC maturation. Significant decreases of CD40 expression were observed when BMDCs activated with LPS were also treated with Ori, whereas the purity and the surface expressions of MHC-II, CD80, and CD86 of DCs were not significantly affected. We also assessed the antigen uptake ability of different groups of BMDCs with fluorescein-bounded ovalbumin (OVA) on FCM. We found no differences between the Ori-treated and control groups (**Supplementary Figure S5**). These results suggested that Ori inhibited *in vitro* BMDC maturation, specifically through CD40 expression. Therefore, Ori treatment may be a potent therapeutic agent in the innate immune response, to reverse DC maturation in solid-organ transplantation.

### Ori-Treated BMDCs Inhibit T-Cell Activation

**Figure 6:**
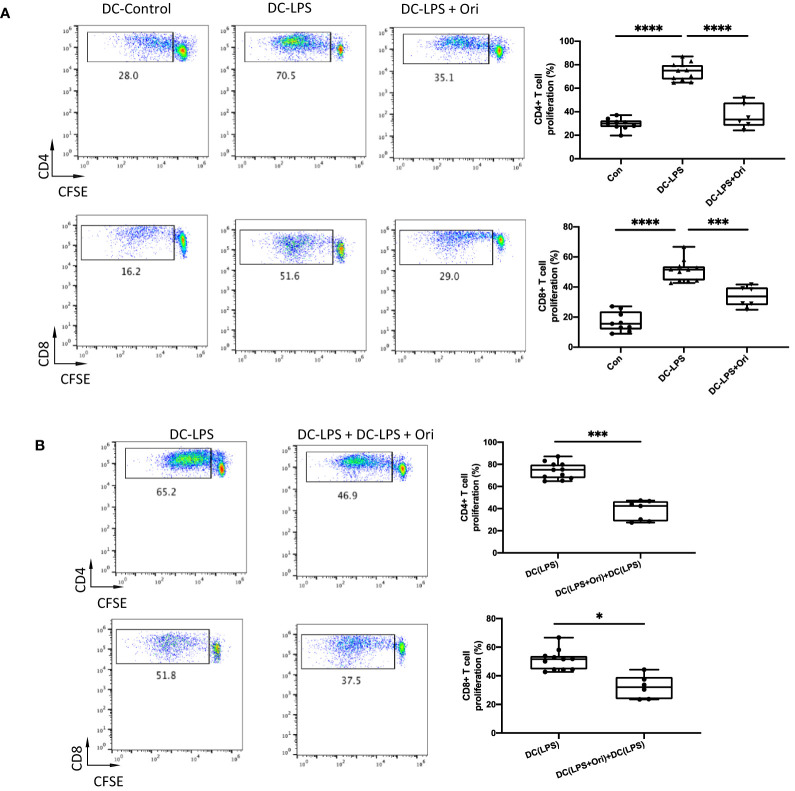
Ori-treated BMDCs could suppress direct T cell proliferative responses *in vitro*. BMDCs were extracted from B6/N and then cultured with IL-4 (10 ng/ml) and GM-CSF (10 ng/ml) *in vitro*. On day 5, different groups were treated with vehicle control; LPS 10 ng/ml (DC with LPS); and LPS (10 ng/ml) and Ori 3 μM (DC with LPS + Ori) for 48 h On day 7, the induced BMDCs (stimulator) were harvested and then irradiated with 20 Gy x-ray. T cells (responder) were isolated from the spleen of C3H, subjected to the Nylon column, and then stained with CFSE for one-way MLR. Coculture the three types of B6 DCs (DC-Vehicle, DC-LPS, and DC-LPS + Ori) with C3H-derived T cells in the ratio of 1:10 for 5 days. In another group, the Ori-treated BMDCs were added at the beginning into the culture as a regulator (regulator:stimulator = 1:1). Proliferation of T cells was stained with CD4 and CD8 and determined by FCM. **(A)** T cells from C3H were cocultured with three groups of BMDCs with the ratio 10:1 for 5 days. Proliferation of Teffs was determined by CFSE gated on CD4^+^ and CD8^+^ T-cell populations. Values are shown as the mean ± SD; *p < 0.05, **p < 0.01. **(B)** T cells from C3H were cocultured with DC-LPS+DC-LPS + Ori (1:1) and DC-LPS with the ratio 10:1 for 5 days. Proliferation of Teffs was determined by CFSE gated on CD4^+^ and CD8^+^ population. Values are shown as the mean ± SD; *p < 0.05, **p < 0.01, ***p < 0.001, ****p < 0.0001.

As Ori downregulated the maturation marker expression in BMDCs activated with LPS, we explored if Ori-treated BMDC function was converted accordingly. It showed that LPS-activated BMDCs remarkably promoted CD4^+^ and CD8^+^ T cell proliferation compared with the imDC control. However, Ori-treated BMDCs abrogated the promoting effects significantly (**Figure 6A**). To identify the direct antigen-presenting regulatory function of Ori-treated BMDCs, we set an MLR group in which an equal quantity of Ori-treated and LPS-activated control BMDCs were co-cultured as stimulators, leading to decreased CD4^+^ and CD8^+^ T-cell propagation (**Figure 6B**). Collectively, these results suggested that Ori promotes BMDC-induced inhibition of T-cell activation *in vitro*.

### Ori-Treated BMDCs Promote Proliferation of Tregs and Suppress Th1 Differentiation

**Figure 7:**
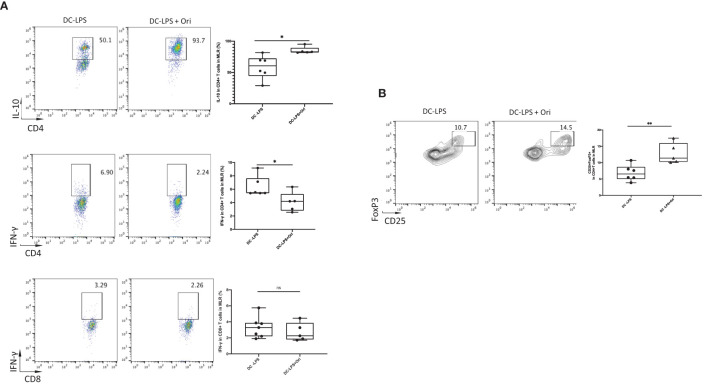
Ori-treated BMDCs could decrease the production of IFN-γ, increased the IL-10, and expanded Tregs *in vitro*. Coculture the two types of B6-derived DCs (DC-LPS and DC-LPS + Ori) (stimulator) with C3H-derived T cells (responder) in the ratio of 1:10 for 5 days *in vitro* for one-way MLR. Then, cells were collected and stained with multiple Abs including the intracellular ones. **(A)** The frequencies of CD4^+^IL-10^+^, CD4^+^IFN-γ^+^ T cells and CD8^+^IFN-γ^+^ T cells. **(B)** Tregs (CD25^+^FoxP3^+^ gated from CD4^+^ T cells) were analyzed by FCM. Representative graphs are given for each group. Values are shown as the mean ± SD; *p < 0.05, **p < 0.01.

To further confirm the mechanisms underlying how Ori-treated BMDCs suppressed the proliferation of T cells, we analyzed the immunomodulatory cytokines and the differentiation of Th cells in the allogeneic MLR *in vitro*. Tregs are the main cells contributing to the induction and maintenance of transplant tolerance. They are differentiated from Th cells in the presence of IL-10 and TGF-β. The results showed that the number of IL-10 secreting CD4^+^ T cells in response to alloantigen was remarkably increased in the Ori-treated BMDC group compared with the LPS-activated group (**Figure 7A**, upper). Moreover, a greater increase in the proportion of Tregs was observed among CD4^+^ T cells following co-culture with Ori-treated BMDCs (**Figure 7B**). The IFN-γ-secreting CD4^+^ T cells were decreased dramatically, suggesting that the Ori-treated BMDCs suppressed Th1-type polarization from naïve T cells. (**Figure 7**, middle). IFN-γ secreting CD8^+^ T cells were noticeably decreased despite not being significantly different (*p* > 0.05; **Figure 7**, lower). The results showed that Ori-treated BMDCs promoted the proliferation of Tregs and suppressed Th1 differentiation *in vitro*, leading to the inhibition of alloreactive T-cell proliferation.

### Ori Treatment Inhibits the Activation of NLRP3 Inflammasomes and the Phosphorylation of NF-κB and IκBα in BMDCs

**Figure 8:**
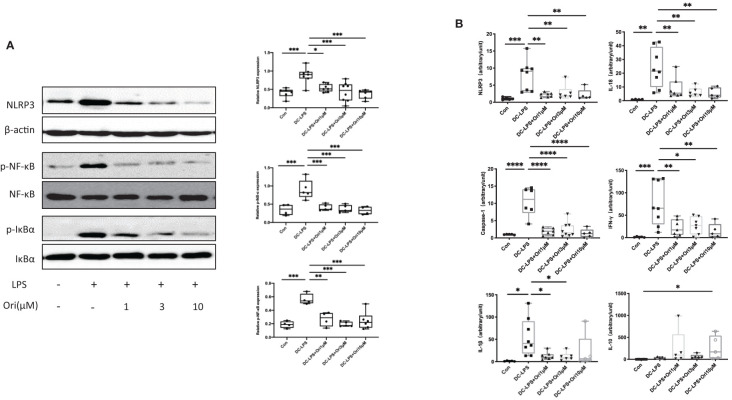
Effects of Ori on the expression of NLRP3/Iκ-B/NF-κB and relative expression of mRNA in BMDCs. BMDCs were induced from bone marrow cells with IL-4 (10 ng/ml) and GM-CSF (10 ng/ml) *in vitro*. Different groups of BMDCs were treated with vehicle control, LPS 10 ng/ml (DC with LPS), and Ori 1, 3, and 10 μM (DC with LPS + Ori) for 48 h Different groups of BMDCs were harvested on Day 7. The expression of protein and mRNA was extracted and measured by Western blotting and qRT-PCR, respectively. **(A)** Western blot analysis of NLRP3, p-Iκ-B, Iκ-B, p-NF-κB, NF-κB, and β-actin proteins in each group. Bar graphs show the relative expression with ratios of NLRP3/β-actin, p-NF-κB/NF-κB, and p-Iκ-B/Iκ-B for each sample. Statistical analysis was determined by one-way ANOVA.*p < 0.05, **p < 0.01, ***p < 0.001, ****p < 0.0001. **(B)** The relative mRNA expression of NLRP3, Caspase-1, IL-1β, IL-18, IFN-γ, and IL-10 normalized with 18S for each sample. Statistical analysis was determined by one-way ANOVA. *p < 0.05, **p < 0.01, ***p < 0.001, ****p < 0.0001.

**Figure 9:**
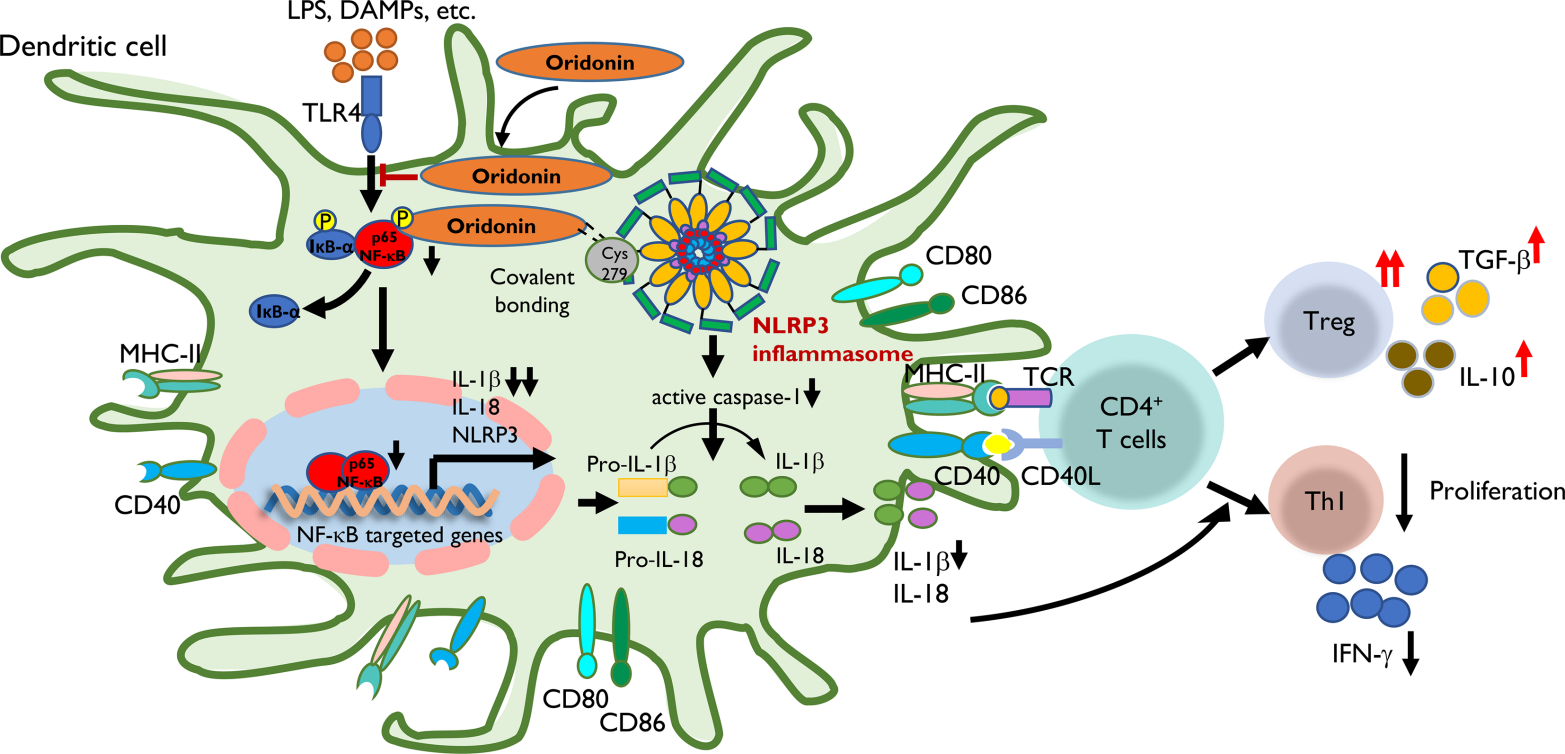
The proposed mechanism by which Ori regulates the transplant immune response through the NLRP3/Iκ-B/NF-κB pathway. Step 1: LPS, DAMPs, etc. were recognized by TLR4 of DC and then induced the transcription of NLRP3, IL-1β, and IL-18 through the Iκ-B/NF-κB pathway with the upregulating expression of MHC-II, CD40, CD80, and CD86. Step 2: Ori could bind to cysteine 279 of NLRP3 *via* covalent bond formation to prevent NLRP3 inflammasome interaction and the subsequent production of IL-1β and IL-18. On the other hand, Ori could interact with the pathway of Iκ-B/NF-κB and prevented its activation. Step 3: In the subsequent immune responses, following the administration of Ori, the activated DC induced the alternation in naïve T-cell differentiation to expand Tregs along with increased cytokines of TGF-β and IL-10, and downregulate Th1 cells with the reduced secretion of IFN-γ. In summary, the functional outcome is to suppress the cellular immune responses.

To address the molecule pathway that Ori targets and affects when suppressing acute cardiac transplant rejection, we investigated the protein and gene expressions of NLRP3 and related proteins in BMDCs. We found that Ori significantly inhibited the activation of NLRP3 and the phosphorylation of NF-κB and IκBα in BMDCs (**Figure 8A**). Consistently, the mRNA expression of NLRP3, Caspase-1, and IL-18 was dramatically downregulated in Ori-treated BMDCs. The expression levels of IL-1β decreased significantly when LPS-activated BMDCs were administrated with 1, 3, and 10 μM Ori (**Figure 8B**). The proinflammatory cytokine IFN-γ was downregulated with all dosages of Ori-treated BMDCs and anti-inflammatory IL-10 was significantly upregulated in 10 μM Ori-treated BMDCs significantly, but not significantly in the 1 μM and 3 μM Ori groups (**Figure 8B**). Collectively, these data demonstrated that Ori prolonged the survival of cardiac allografts through inhibiting Th1 proliferation and promoting Treg development, dependent on the attenuated NF-κB/NLRP3 signaling pathway (**Figure 9**).

## Discussion

Ori is a crucial bioactive ingredient in *R. rubescens*, an over-the-counter herbal medicine widely used for inflammatory diseases (42). Ori exerts antitumor activity through the regulation of the expressions of ERK, Bax/Bcl-2, and NF-κB in various cancer diseases, such as leukemia, breast cancer, gastric cancer, and lung cancer (42, 43). Many researchers also focus on its anti-inflammatory effects, shown to exhibit certain therapeutic efficacy on neuroinflammation, sepsis, and colitis through suppressing the activation of MAPK or NF-κB and inhibiting the release of some proinflammatory cytokines, such as TNF-α and IL-6 (15, 44). Previously, Ori was shown to prolong the survival of skin allograft through promoting T-cell apoptosis and depletion of T cells in peripheral blood and spleen in a time- and dosage-dependent manner (37). We aimed to delineate the protective effect of Ori on solid organ allograft survival and elucidate its immunomodulatory mechanism. Different dosages of Ori (3, 10, and 15 mg/kg) were tested in a mouse allogeneic cardiac transplantation model. Our results showed that Ori with 3, 10, and 15 mg/kg Ori could prolong the survival (MST = 22.8, 49.2, and 65.3 days, respectively) significantly compared with the control group (MST = 8 days; **Figure 1**). Although no obvious toxicity or side effects were observed in the traditional use of Ori, mice treated with 15 mg/kg Ori showed dull, anorexic, and unkempt features, 3 days post-operation. The safety and cytotoxicity of Ori require further investigation. Considering the IS activity and toxicity of the administration of Ori, we chose the dosage of the 15 mg/kg model *in vivo* to decipher its underlying suppressive mechanism.

The NLRP3 inflammasome, an intracellular signaling complex that activates potent inflammatory cascade responses, is relevant in numerous infectious and autoimmune diseases like peritonitis, sepsis, T2D, systemic lupus erythematosus, and rheumatoid arthritis (21, 29, 30). NLRP3, the key sensor in the inflammasome, recognizes a variety of DAMP and PAMP antigens such as microbes, viral RNA, extracellular ATP, ROS, and damaged lysosomes (19, 21, 23, 29). The eventual reassembly and activation of the inflammasome leads to proptosis and the release of inflammatory cytokines including IL-1β and IL-18 and chemokines, contributing to the differentiation and proliferation of proinflammatory T cells and tissue damage (20). As a result, NLRP3 is an attractive and promising drug target for inflammatory-related immune responses. He et al. reported that Ori binds to cysteine 279 of NLRP3 covalently and inhibits the activation of the NLRP3 inflammasome specifically (16). Thus, we hypothesized that Ori treatment may exert IS effects on mouse cardiac allografts by inhibiting the NLRP3 inflammasome signaling pathway.

We found that Ori reduced the total inflammatory infiltration, specifically CD8^+^ T-cell and macrophage infiltration in allografts and expanded the percentage of Tregs on POD7. It also downregulated IL-1β and IFN-γ and upregulated TGF-β expression *in vivo* (**Figures 2** **–** **4**). We subsequently investigated the effects of Ori on DC and the differentiation of T cells *via* the culture of BMDC and the setting of alloreactive MLR *in vitro.* We found that Ori partially inhibited BMDC maturation, mainly by regulating CD40 expression (**Figure 5**). It also downregulated the gene expressions of NLRP3, Caspase-1, IL-1β, IL-18, and IFN-γ and upregulated the expression of IL-10 (**Figure 8B**). Ori inhibited NLRP3 expression and attenuated the phosphorylation of NF-κB and IκBα in LPS-activated BMDCs (**Figure 8A**). In the MLR assay, Ori-treated BMDCs suppressed the proliferation of CD4^+^ and CD8^+^ T cells and the differentiation of IFN-γ^+^CD4^+^ T cells, along with the expansion of Tregs and IL-10^+^CD4^+^ T cells (**Figure 7**). These results indicated that Ori prolonged the survival of allogeneic cardiac allograft by targeting NLRP3 to exert its immunosuppression activity on acute rejection.

Based on our results, Ori prolonged the survival of cardiac allograft *via* regulating the allogeneic immune response through attenuating the NLRP3/Iκ-B/NF-κB pathway in DCs (**Figure 9**). Alloantigens including LPS and DAMPs can be recognized by TLR4 of recipient DCs and then induce the transcription of NLRP3, IL-1β, and IL-18 through the Iκ-B/NF-κB pathway by upregulating expression of MHC-II, CD40, CD80, and CD86. Ori prevented the activation of the NLRP3 inflammasome *via* covalent bonding to cysteine 279 of NLRP3 and interacted with the Iκ-B/NF-κB pathway, preventing its activation in recipient DCs, following the downregulation of IL-1β and IL-18. Subsequently, the Ori-treated DCs induced the alternation in naïve T-cell differentiation to expand Tregs along with increased cytokines of TGF-β and IL-10 and downregulated the Th1 cells with the reduced secretion of IFN-γ. Collectively, the functional outcome suppressed the cellular immune responses.

Several studies have focused on the mechanism of NLRP3 inflammasome function in adaptive immune responses (26–28, 33). Bruchard et al. reported that NLRP3 acts as a transcription regulator in CD4^+^ T cells and promotes Th2 cell differentiation in asthma and melanoma models (27). Arbore et al. demonstrated that the NLRP3 inflammasome of CD4^+^ T cells promotes the release of IFN-γ and the differentiation of Th1 and Th17 *via* the autocrine IL-1β in the autoimmune colitis and GvHD models (28). Amores-Iniesta et al. found that extracellular ATP released from donor immune cells could be recognized by the P2X7 receptor in macrophages in a mouse skin transplantation model, subsequently activating the NLRP3 inflammasome and promoting IL-18 maturation, resulting in Th1 and CD8^+^ T-cell proliferation and decreased Treg and IFN-γ production (26). Wei et al. revealed that the activated NLRP3 inflammasomes exacerbated allograft rejection in a corneal transplantation model by promoting IL-1β production and Th17 differentiation by amplifying the mechanistic STAT3 phosphorylation (33). Consistently, we also demonstrated that Ori, as a specific mediator of NLRP3, could reduce the assembly and activation of the NLRP3 inflammasome and the release of IL-1β and IL-18. It subsequently decreased T-cell proliferation and expanded Treg in a cardiac transplant model. Paradoxically, we examined the percentage of CD4^+^IL-17^+^ cells in the MLR without significant difference (data were not shown). In our study, the DAMPs following allograft were recognized by TLR4 on the surface of DCs in a classical manner and then activated the signaling pathway of NF-κβ, playing a priming role for NLRP3 in the inflammasome. Except for the covalent inhibitor of NLRP3, Ori can target NF-κB activation (13, 45). Thus, we speculated that Ori could exert IS activity in acute allograft response through the NLRP3 and NF-κB pathways synergistically, whereas the mRNA levels of NLRP3 in GILs and SPCs were not altered significantly in the Ori-treated group, and even the tendency of the mRNA level in SPCs was ascending compared with the control. Consider the spleens and GILs to be a mixture of various immunocytes, in which some specific cells like monocytes, macrophages, and neutrophils could be activated and increase the total expressions of NLRP3. In this study, we mainly aimed at the effects of Ori on DCs and its regulation of T cells; thus, the results of NLRP3 and relevant molecules *in vitro* were used to illustrate the potential effects of Ori.

However, we mainly examined the expression of NLRP3 and related molecules in BMDCs treated with Ori *in vitro*, without examining extracellular ATP, the P2X7 receptor, and the Th2 or Th17 differentiation; thus, the results might not be integrated. Additionally, we just investigated histological and molecular changes about acute rejection on POD7 in this study; however, owing to the lack of experiments on longer time points, the immune status of Ori-treated recipients, the persistence of infiltrated DCs and Tregs, or the expression of NLRP3 and other molecules could not be confirmed as delayed acute rejection, chronic rejection, antibody-mediated rejection, or real transplant tolerance, and the present results were still kind of inadequate. Thus, further experiments addressing these issues and clarifying the underlying mechanisms of Ori in cardiac allograft are warranted.

This is the first study to focus on the effect of Ori on allograft rejection by inhibiting the NLRP3 pathway. Our findings confirmed that Ori prolonged the allograft survival and exerted IS activity *via* inhibiting the NF-κB/NLRP3 signaling pathway. It also decreased IL-1β and IL-18 production and Th1 differentiation and proliferation and expanded Tregs in our mouse cardiac transplantation model. Our findings highlight Ori as a novel and natural therapeutic agent for promoting real transplant tolerance without paralyzing the entire immune system.

## Supporting Information

Additional supporting information may be found online in the Supporting Information section at the end of the article.

## Data Availability Statement

The raw data supporting the conclusions of this article will be made available by the authors, without undue reservation.

## Ethics Statement

The animal study was reviewed and approved by Animal Use and Care Committee of the National Research Institute for Child Health and Development, Tokyo, Japan.

## Author Contributions

XD, WQ, XH, XY, W-ZG, SZ, and X-KL conceived and designed the project. XD and WQ acquired the data. XD, WQ, XH, XY, W-ZG, SZ, and X-KL analyzed and interpreted the data. XD and X-KL wrote the paper. All authors contributed to the article and approved the submitted version.

## Funding

This study was supported by research grants from the National Nature Science Foundation of China (81671958 and U1604282); Science and Technology Innovation Talents in Henan Universities (No. 19HASTIT003); Grants of Ministry of Education, Culture, Sports, Science and Technology of Japan (Grants-in-Aid 16K11064, 24/17H04277, and 18K08558); and grants from the National Center for Child Health and Development (29–09).

## Conflict of Interest

The authors declare that the research was conducted in the absence of any commercial or financial relationships that could be construed as a potential conflict of interest.

## Publisher’s Note

All claims expressed in this article are solely those of the authors and do not necessarily represent those of their affiliated organizations, or those of the publisher, the editors and the reviewers. Any product that may be evaluated in this article, or claim that may be made by its manufacturer, is not guaranteed or endorsed by the publisher.
