# Supplementary material for: Oridonin Prolongs the Survival of Mouse Cardiac Allografts by Attenuating the NF-κB/NLRP3 Pathway
Source: Front Immunol. 2021 Sep 10;12:719574. doi: 10.3389/fimmu.2021.719574 (PMC8462485; doi:10.3389/fimmu.2021.719574)
Supplement: Supplementary file 1 [file DataSheet_1.docx]

Supplementary Material

**1. Supplementary Materials and Method**

**1.1 Flow cytometry (FCM)**

Graft-infiltrating lymphocytes (GILs) was obtained from heart graft after cutting into 1~2mm pieces, digesting with 0.5 mg/ml collagenase IV (Sigma-Aldrich, Saint Louis, MO) and 50 U/ml DNase (Thermo Fisher, Waltham, MA) in complete media at 37℃ for 20 minutes, and then isolating with Lympholyte-M (Cedarlane, Ontario, Canada). Splenocytes (SPCs) were obtained from spleens of recipient mice after grinding the spleen and lysing the erythrocytes. After Fc block with CD16/32 and live/dead staining (Thermo Fisher), SPCs and GILs were labeled for surface antigens with anti-mouse conjugated anti-CD45 (30-F11), anti-CD3 (145-2C11), anti-CD4 (GK1.5), anti-CD8 (53-6.7), anti-B220 (RA3-6B2), anti-Nkp46 (29A1.4), anti-CD11b (M1/70), anti-CD11c (N418), anti-Ly6C (HK1.4), anti-Ly6G (1A8), anti-Nrp1 (3E12), anti-CD44 (IM7), anti-CD62L (MEL-14) mAb, anti-CD69(FN50)，anti-CD103 (2E7); for the DC in vitro , cells were stained with anti-CD11b (M1-70), anti-CD11c (N418), anti-MHC-II (M5/114.15.2), anti-CD40 (3/23), anti-CD80 (16-10A1) and anti-CD86 (GL-1) conjugated with a particular fluorochrome. Cells from mixed lymphocyte reaction (MLR) were stained with CD4-APC (RM4-5) and CD8a-AF700 (53-6.7). The relevant isotype controls were used (BioLegend, San Diego, CA). After the surface mAbs staining, intracellular staining for FoxP3 was performed with anti-FoxP3 (MF-14) mAb.

For intracellular cytokine staining, cells from MLR were stimulated with a leukocyte activator cocktail, include 50 ng/ml PMA (BD Golgi Plug™), 1 μM ionomycin (Sigma-Aldrich) and Brefeldin A (eBioscience, San Diego, CA) in complete medium for 4 hours in a 37℃, 5% CO2 incubator. Subsequently, after performing the above surface mAbs staining protocol and fixation/permeabilization, stained with IL-10-PE (JES5-16E3) and IFN-γ-PE/Cy7 (XMG1.2) (BioLegend). The analyses of stained cells were conducted with LSR Fortessa cytometry (BD Biosciences) and SP6800 spectral cell analyzer (Sony, Tokyo, Japan), and these data were analyzed via the FlowJo (Version 10.7.0; BD Biosciences).

**1.2 Histological analysis**

Cardiac grafts were obtained on POD7 and fixed in 10% formalin, dehydrated, embedded in paraffin, cut into 4-μm thickness slices and then stained with hematoxylin and eosin. Acute graft rejection was graded on the extent of inflammatory infiltration and myocardial damage according to the International Society of Heart and Lung Transplantation (ISHLT) grading criteria1 as follows: 0, no rejection; 1, mild rejection with up to 1 focus of inflammatory infiltration and myocyte damage; 2, moderate rejection with two or more focus of inflammatory infiltration and myocyte damage; 3, severe rejection with diffuse infiltration and myocyte damage or with edema, hemorrhage and vasculitis.

**1.3 Immunohistochemistry**

Cardiac grafts were obtained on POD7, embedded in Tissue-Tek O.C.T compound (Sakura Finetek, Torrance, CA), frozen rapidly in -80°C Hexane (Wako, Osaka, Japan) and then cut into 4-μm thickness cryosections. Double immunostaining was conducted. After being rehydrated and fixation with Formaldehyde calcium solution, sections were blocked with using block ace for 10 minutes. The rat anti-mouse CD4, CD8α, CD68 and F4/80 mAbs (Biolegend) were used as the primary antibodies separately. Sections were incubated with primary antibody working solution (1:100 dilution) at room temperature for 1 hour. ALP-conjugated donkey anti-rat IgG (Jackson ImmunoResearch, West Grove, PA) was used as secondary antibody with 1:100 dilution at room temperature for 1 hour. For visualizing the positive antigens, Vector® Blue Alkaline Phosphatase (Blue AP) Substrate Kit (VECTOR Laboratories, Burlingame, CA) was used following its protocol.

Subsequently, after washing adequately, type IV collagen staining was performed. All sections were incubated with rabbit-anti-mouse type IV collagen polyclonal Ab (Cosmo Bio, Tokyo, Japan) for 1 hour. POD-conjugated goat-anti-rabbit Ig (Jackson ImmunoResearch,) was used as secondary Ab incubating for 1 hour and then developed the DAB (Dojindo, Kumamoto, Japan) substrate reaction. Ultimately, sections were fixed in Formaldehyde calcium solution and mounted with Aquatex (Sigma Aldrich). All images were captured by a DP70 camera (Olympus, Osaka, Japan) and then the ratio of positive cells in total was analyzed quantitatively with the WinRoof software (V.6.1; Mitani Corporation, Tokyo, Japan)2, 3. For each slide, the ratio of positive cells was evaluated in 5 non-overlapping fields each at original magnification x400.

**1.4 Quantitative real time RT-PCR**

Total RNA from cardiac grafts was extracted using reagent Sepasol®-RNA I Super G (NACALAI TESQUE, Inc., Kyoto, Japan). RNA from SPCs and DCs was isolated by using the RNeasy Mini kit (Qiagen, Valencia, CA) according to the manufacturer's protocol. After the removal of the potential DNA inside with the DNA-freeTM Kit (Thermo Fisher), total RNA was reverse transcribed for generating cDNA by using the PrimeScript RT reagent Kit (Takara Bio, Shiga, Japan). Quantitative real-time PCR analysis was carried out by using the ABI 7900HT Sequence Detection System (Thermo Fisher) with the SYBR system. The sequences of 18s and target gene primers used in this research were shown in Table S1. The analyses of the expression of target genes were calculated by subtracting the Ct value of 18s rRNA and using the 2-ΔΔCt method.

**1.5 Western Blotting**

Groups of BMDCs were harvested on Day 7, washed third times with PBS and incubated with RIPA lysis buffer (Wako) containing 1% protease inhibitor cocktail, 1% phosphatase inhibitor cocktail 1 and 1% phosphatase inhibitor cocktail 2 (Sigma-Aldrich) for 20 minutes on ice. After centrifuging the lysis samples at 16,300 g for 20 minutes, the protein supernatants were collected and measured concentrations with BCA Protein Assay (Thermo Scientific). Twenty μg of proteins were processed with SDS-PAGE electrophoresis and transferred onto PVDF membranes (Bio-Rad, Hercules, CA). The membranes were blocked with 5% BSA in TBS for 1 hour at room temperature, and then incubated with the primary antibodies containing anti-NF-κB p65 (1:1000), anti-pNF-κB p65 (1:1000), anti-IκB-α (1:2000), anti-pIκΒ-α (1:2000) (Cell Signaling Technology (CST), Danvers, MA), anti-NLRP3 (Cryo-2) (1:2000, AdipoGen Life Sciences, San Diego, CA) and β-actin (1:2000, CST) separately overnight at 4°C. After washing three times with TBST, the membranes were probed with HRP-conjugated anti-mouse IgG (1:3000, CST) or HRP-linked anti-rabbit IgG (1:3000, CST) secondary antibodies for 1 hour at room temperature. Chemiluminescence signal intensity of protein bands were subjected to the enhanced chemiluminescence (ECL), visualized by ImageQuant LAS4000 System (GE Healthcare, Little Chalfont, UK) and then quantitated with Image J software normalizing to that of β-actin.

**1.6 Reference**

1. Stewart S, Winters GL, Fishbein MC, et al. Revision of the 1990 Working Formulation for the Standardization of Nomenclature in the Diagnosis of Heart Rejection. The Journal of Heart and Lung Transplantation. 2005;24(11):1710-1720. doi:10.1016/j.healun.2005.03.019

2. Hatanaka Y, Hashizume K, Nitta K, Kato T, Itoh I, Tani Y. Cytometrical image analysis for immunohistochemical hormone receptor status in breast carcinomas. Pathol Int. Oct 2003;53(10):693-9. doi:10.1046/j.1440-1827.2003.01547.x

3. Hayashi T, Morishita E, Ohtake H, Oda Y, Asakura H, Nakao S. Expression of annexin II in experimental abdominal aortic aneurysms. Int J Hematol. Oct 2009;90(3):336-342. doi:10.1007/s12185-009-0410-6.

**2. Supplementary Figures and Tables**

**2.1 Supplementary Figures**


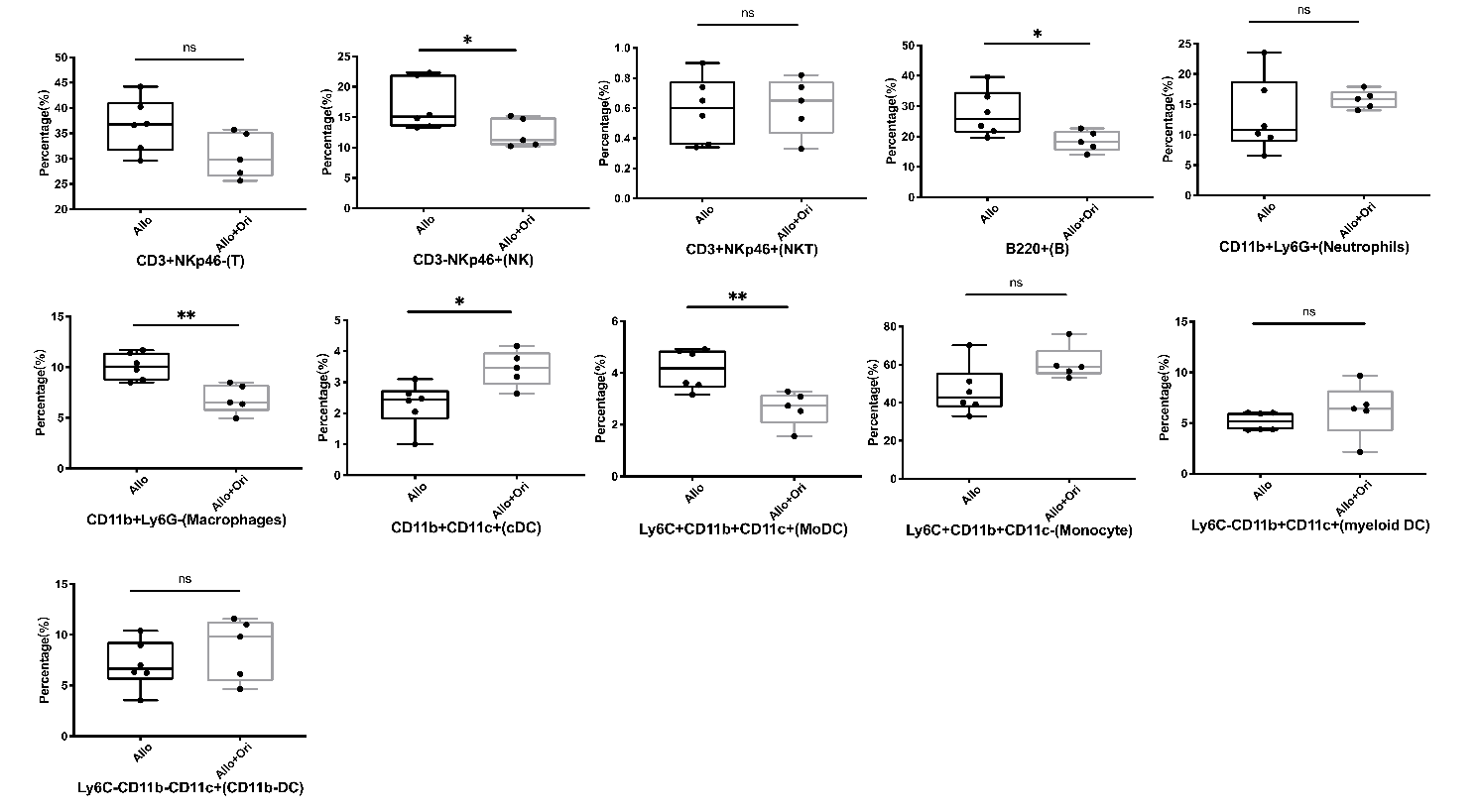


# Supplementary Figure 1. The effects of Ori on the different populations of immune cells in spleen. Spleens were harvested on POD7 from control (Allo) group and Ori-treated (Allo + Ori) group. Graphs depict individual values and group mean ± SD. Data were analyzed with t-test. *p<0.05, **p<0.01, ns, not significant.


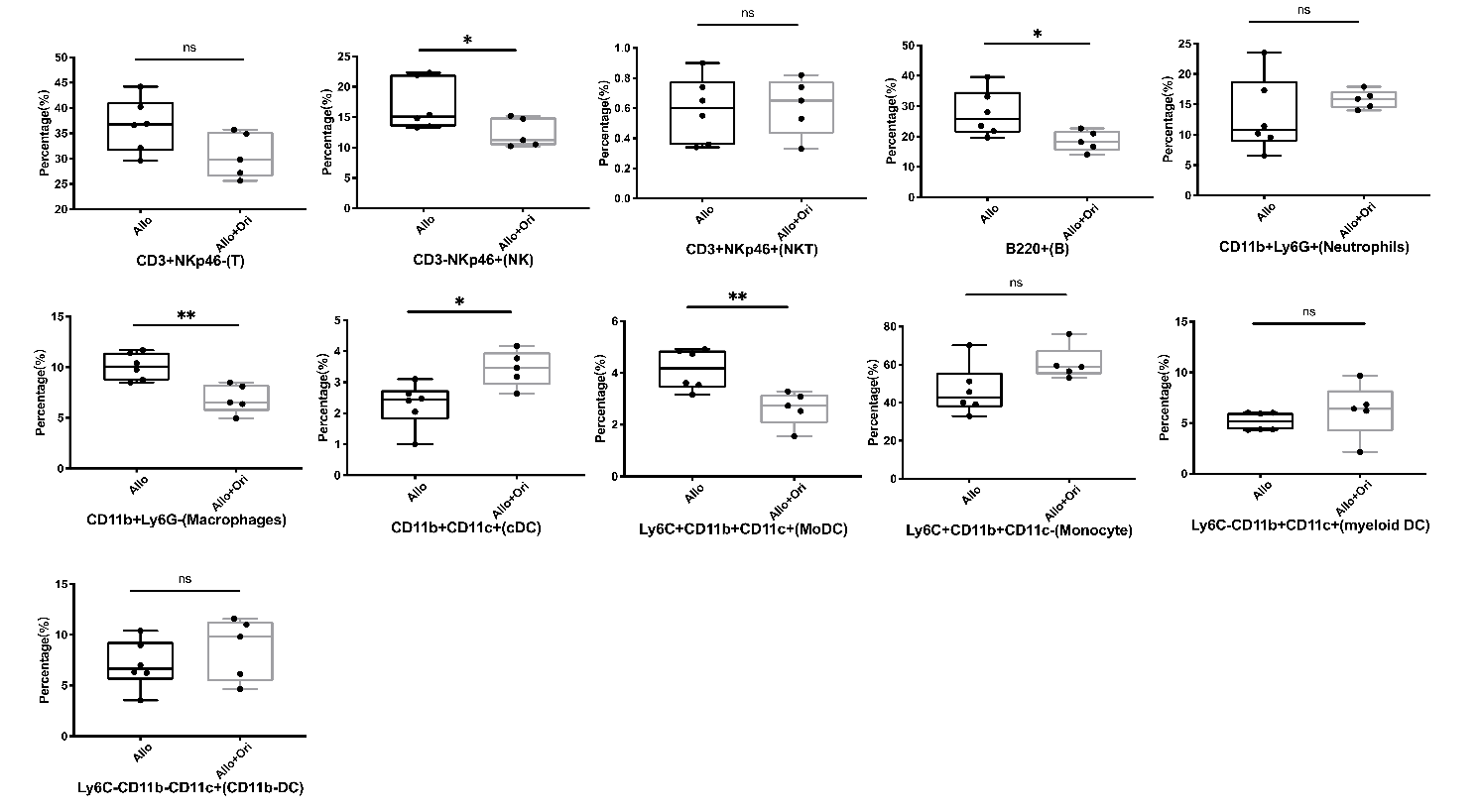


# Supplementary Figure 2. The effects of Ori on the different populations of immune cells in GILs. Cardiac grafts were obtained on POD7 from control (Allo) and Ori-treated (Allo + Ori) groups. GILs were isolated from graft, multiply stained with mAb and then assessed by FCM. Graphs depict individual values and group mean ± SD. Data were analyzed with t-test. *p<0.05, **p<0.01, ns, not significant.


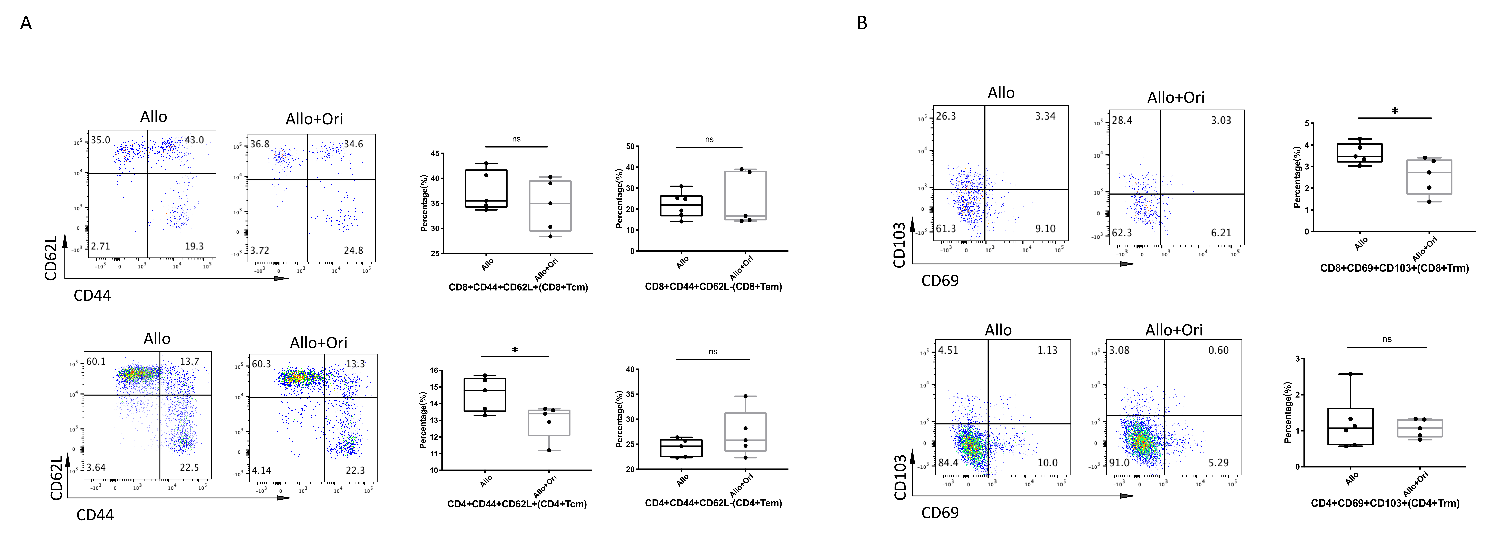


# Supplementary Figure 3. The effects of Ori on central and effector memory T cells (TCM and TEM) and tissue-resident memory T cells (TRM) in SPCs. The representative plots of TCM(CD44+CD62L+), TEM (CD44+CD62L-) and TRM (CD69+CD103+) cells in the CD4+ and CD8+ T cells are presented. The data were shown as the mean ± SD, data were analyzed with Student’s t-test. *p<0.05, ns, not significant.


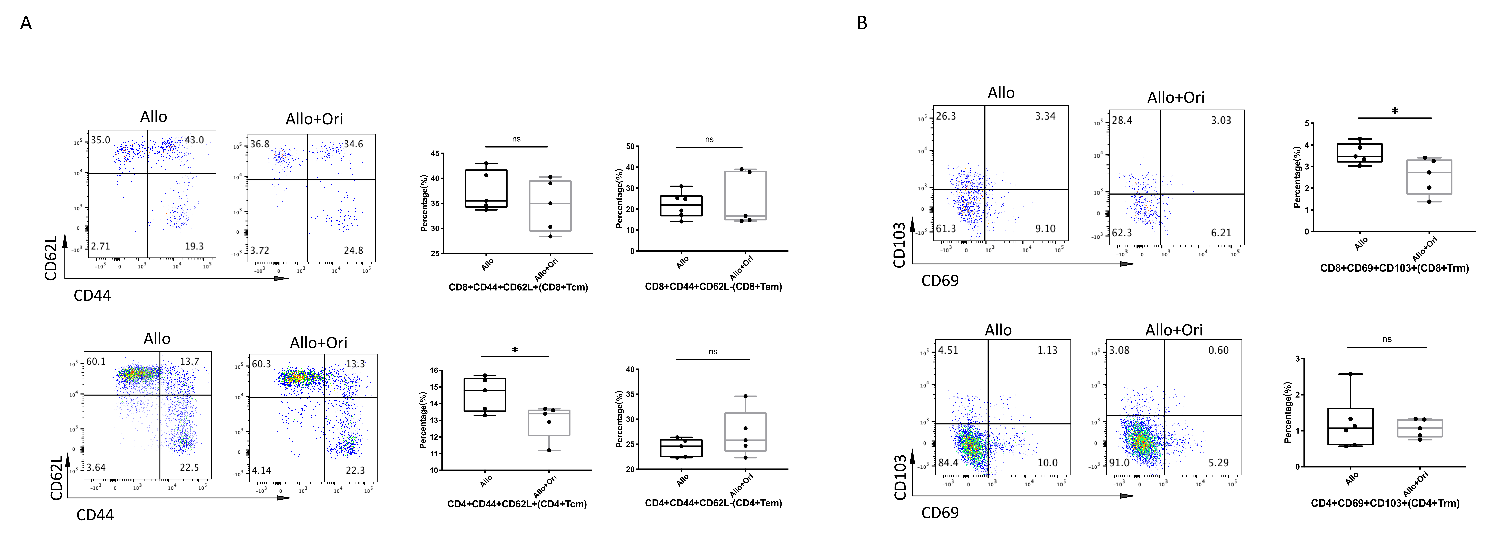


# Supplementary Figure 4. The effects of Ori on the central and effector memory T cells (TCM and TEM) and tissue-resident memory T cells (TRM) in GILs. The representative data of TCM (CD44+CD62L+), TEM (CD44+CD62L-) and TRM (CD69+CD103+) cells in the CD4+ and CD8+ T cells are presented. The data were shown as the mean ± SD, data were analyzed with Student’s t-test. ns, not significant.


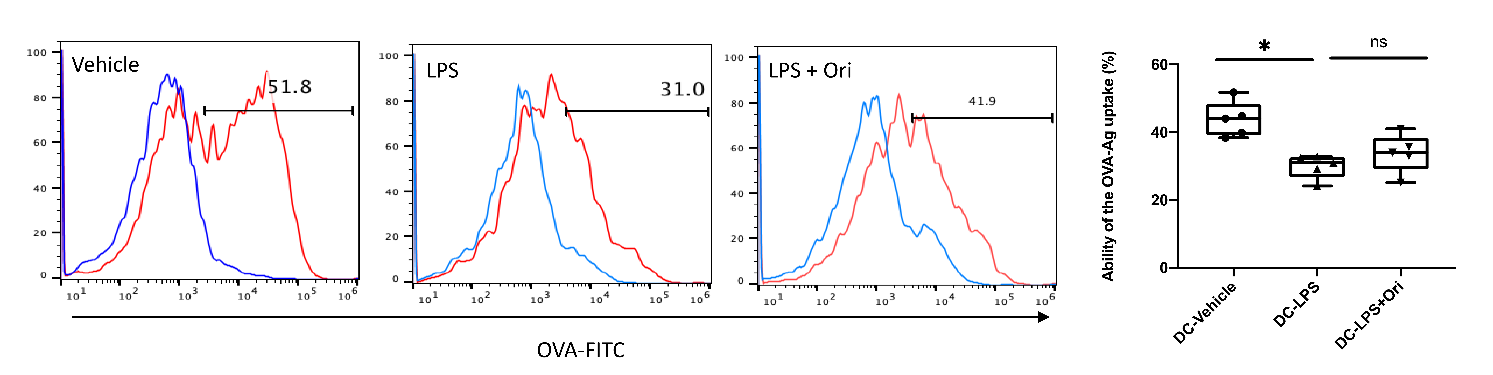


# Supplementary Figure 5. Antigen uptake ability test of BMDCs after Ori-treatment on Day 7. Different groups of BMDC were treated with negative control (imDC), LPS 10ng/ml (DC with LPS) and Ori 1, 3, 10 μM (DC with LPS + Ori) for 48h and harvested on Day 7. Antigen uptake ability of BMDCs for protein antigen (OVA-FITC) was detected by FCM. The blue line means the antigen uptake ability of DCs on 4℃, and the red line means the antigen uptake ability on 37℃. The differences between 37℃ and 4℃ were statistically analyzed one-way ANOVA. *p< 0.05, ns: not significant.

**2.3 Supplementary Tables**

| Table 1. Primer sequences for real-time PCR. | | |
| --- | --- | --- |
| Gene | Forward Primer (5'~3') | Reverse Primer (3'~5') |
| NLRP3 | TGTGTGGATCTTTGCTGCG | GGAATGTGATGTACACGTGTCATTG |
| Caspase-1 | ACCACTCGTACACGTCTTGC | TGGGCAGGCAGCAAATTCTT |
| IL-1β | ACCTTCCAGGATGAGGACATGA | AACGTCACACACCAGCAGGTTA |
| IL-10 | GGGTGAGAAGCTGAAGACCCT | TCACCTGCTCCACTGCCTT |
| IL-18 | AGCAGTCCCAACTAAGCAGTA | CAGCCAGTAGAGGATGCTGA |
| TGF-β | AGGTCACCCGCGTGCTAA | CACACTTCGACGGATGAGATCA |
| TNF-α | TGTCTACTGAACTTCGGGGTGAT | AACTGATgAGAGGGAGGCCAT |
| IFN-γ | AAGCGTCATTGAATCACACCTGA | ACCTGTGGGTTGTTGACCTCAA |
| 18S | ACATCGACCTCACCAAGAGG | TCCCATCCTTCACATCCTTC |
